# Treatment of Fracture Sequel in the Mandibular Angle Region

**DOI:** 10.1155/2019/4627301

**Published:** 2019-07-04

**Authors:** Beatriz Sobrinho Sangalette, Tiago Trevizan Levatti, Larissa Vargas Vieira, João Lopes Toledo Filho, Cláudio Maldonado Pastori, Gustavo Lopes Toledo

**Affiliations:** ^1^Marília University-UNIMAR, Av. Higino Muzi Filho, No. 1001, CEP, 17525-902 Marília, SP, Brazil; ^2^Departmente of Biological Science-Anatomy, Bauru School of Dentistry, University of São Paulo-FOB/USP, Rua Alameda Dr. Octávio Pinheiro Brisolla, No. 9-75, CEP, 17012-901 Bauru, SP, Brazil; ^3^Department of Bucomaxilofacial Surgery, University Center of Adamantina-UNIFAI, Av. Francisco Bellusci, No. 1000, CEP, 17800-000 Adamantina, SP, Brazil; ^4^Department of Oral Maxillofacial Surgery, State University of North Paraná (UENP), Av. Getúlio Vargas, No. 850, CEP 86400-000, Jacarezinho, PR, Brazil

## Abstract

Because of the anterior disposition on the face and the fragility of the anatomy, the mandible is commonly affected in facial fractures, and the angle region represents 32% of the mandibular fractures; therefore, the objective of the paper was to present a proposal for late correction of the mandibular fracture already consolidated and with occlusal alteration. Patient J.C.P.R., 32, during anamnesis reported loss of sensibility in the mentalis region as well as unilateral posterior open bite for having been a victim of an automobile accident about 1 year and 3 months ago. Physical examination showed elevation in the right mandibular angle region due to the poor positioning of fractured stumps. It has been found that the patient suffered a simple fracture at the mandibular angle, but that had not been treated previously, and it was necessary to treat the fracture sequel. Refraction was performed with a new reduction and fixation through titanium plates and screws, showing that, even being late, the procedure to reduce the fracture sequela was effective, even with the correct functional occlusal adjustment.

## 1. Introduction

The mandibular bone represents a large percentage of fractures resulting from trauma, the main causes of which are found in the literature, automobile accidents and physical aggression [1–3]. This fact may be related to the anterior position that the mandible occupies in relation to the other bones of the face and its own anatomy, divided from the anterior to a posterior plane, respectively, in the symphysis region, parassifinise, body, alveolar process, angle, branch, and coronary and condylar apophysis [4].

From the aforementioned, the mandibular angle region represents 32% of the facial traumas and, considering the complexity of the lesions presented by the patient, when this one demonstrates more than one, the reduction and fixation of these should be performed as soon as possible, in order to obtain the correct positioning of the fractured stumps, thus avoiding the sequelae [5].

This report will describe the surgical treatment of the correction of the fracture sequela located in the region of the right mandibular angle, demonstrating that this denotes viability when following the correct criteria of functional occlusal reduction.

## 2. Case Presentation

The patient J.C.P.R., male, leucoderma, 32 years old, appeared at the Outpatient Clinic of Buccomaxillofacial Surgery and Traumatology of the Hospital Beneficência Portuguesa of Bauru/São Paulo/Brazil, with the main complaint of unilateral posterior open bite, limitation of mouth opening amplitude, and sensibility loss in the mentalis region. During the medical-dental questioning, he reported having suffered a car accident about one year and three months ago, which motivated the fracture, generating painful discomfort, being this symptom relieved by the continuous use of analgesics and anti-inflammatories, delaying the search for a treatment. He denied visual and respiratory alterations.

**Figure 1:**
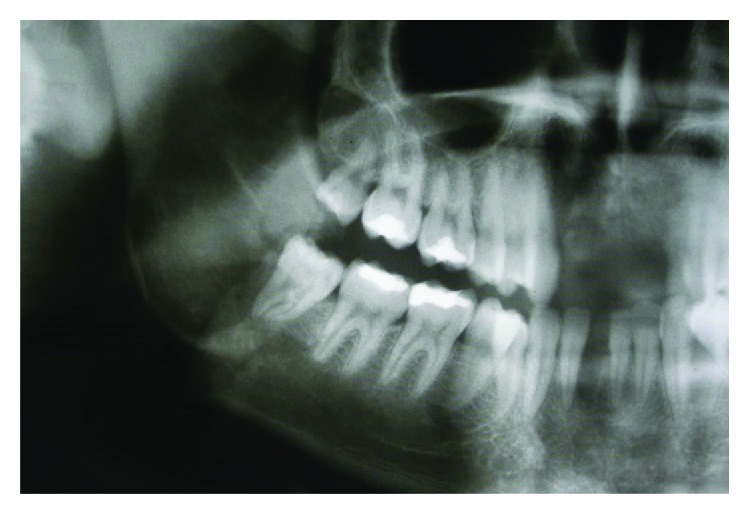
Orthopantomography demonstrating incorrect union of the fractured extremities in the right quadrant mandibular angle.

Physical examination revealed an increase in volume in a region of the right mandibular angle, in addition to a misalignment of the mouth without evident signs of fever or inflammatory process, which indicates the possible pseudoconsolidation of the fracture. In the radiographic examination, we observed an overlap of the fractured stumps in the same area with the absence of other sequelae (Figure 1). It has been seen that the patient had suffered a simple fracture at the mandibular angle, however without any reduction; therefore, a sequela treatment was needed.

### 2.1. Treatment

**Figure 2:**
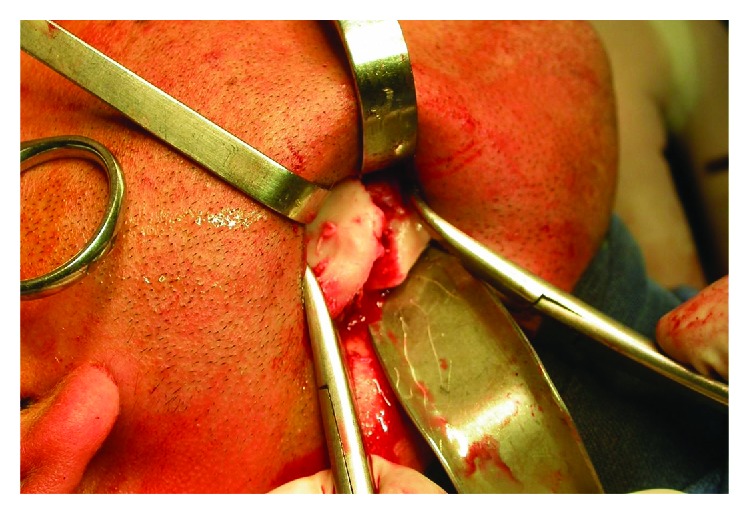
Exposure of the refracted right mandibular angle.

The patient was in HDD, submitted to general anesthesia, and performed intra- and extraoral antisepsis with degermant and topical Pvpi, placed in sterile fields. After marking, the incision in the Risdon approach with blade number 15 and divulsion in planes with Metzenbaum scissors began. When the facial nerve was located, the same was gently moved away to be protected. When the pterigomasseteric band was located, it was incised and detached with molti; the detachment extended over the entire length of the fracture until the complete view of the lingual plate. With the help of a chisel and a hammer, the locks were released (Figure 2).

**Figure 3:**
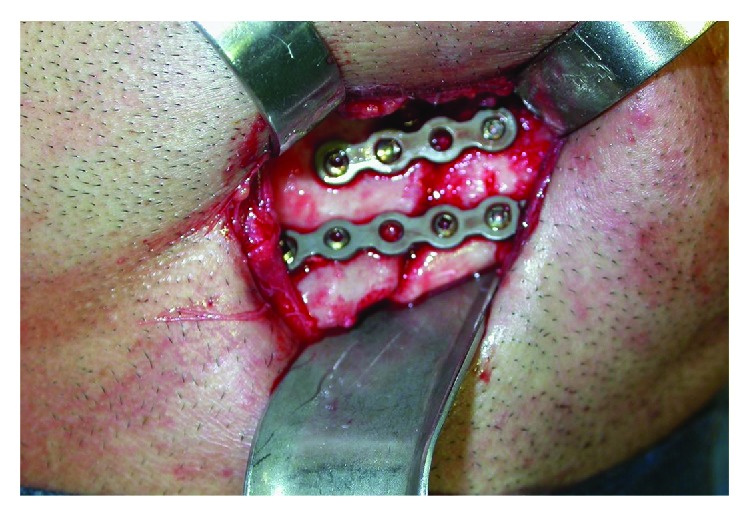
Rigid fixing with titanium plates and screws.

There was no sign of osteomyelitis or obvious inflammatory processes. An osteoid mass of disorganized tissue was found and easily removed. Among the stumps, vascularization was observed within the norms of normality, which certainly favored bone repair. The insertion of Erich's bar and intermaxillary block was performed, obeying the functional occlusion. Again, in the Risdon approach, the fracture was reduced after ostectomy, removal of the osteoid mass. It was fixed with titanium plates and screws (Figure 3).

The adjacent musculature was sutured and finalized with 6.0 mononylon. In the 7-day postoperative period, it had good cicatricial appearance, stitches were in position, there was an absence of inflammatory signs, there was occlusal stability, and the patient denied painful discomfort, demonstrating that, even late, the procedure for reducing the fracture sequel is effective including the correct occlusal adjustment.

### 2.2. Outcome and Follow-Up

**Figure 4:**
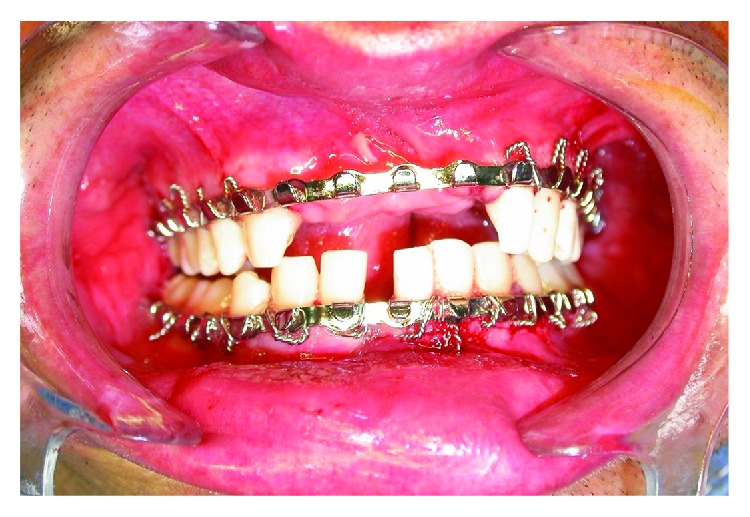
Seven-day postoperative period showing functional occlusion correction.

The patient followed a postoperative follow-up for 7, 14, 21, 35, and 64 days, presenting evolution within normality patterns (Figure 4). There was an absence of secondary infections, and there was occlusal restoration. However, due to the fact that he had sought the service after one year and three months, it was not possible to reverse the paraesthesia.

## 3. Discussion

The treatment of mandibular fractures has evolved considerably over the decades, going through several advances since the advent of the Hippocratic concept of rapprochement and immobilization [1]. The literature refers to automobile accidents and physical aggression as being the main causes of mandibular fractures [4], agreeing with the present report which brings etiology of automobile trauma.

Among the subdivisions of the mandible, the angle region concentrates about 20% to 30% of all mandibular traumas, and its reduction and fixation are procedures that must be performed immediately/mediated, taking into account the severity and complexity of other injuries presented by the patient [6, 7]. The late treatment, presented in this study, may cause clinical complications, which in the majority will become irreversible leading to undesirable disorders, which will last throughout the patient's life, to exemplify, the clinical picture of paresthesia of the mental nerve, in addition to sensitized regions by the inferior alveolar nerve, besides the appearance of infections of difficult treatment in an unpredictable course, which was not the case in the presented report [7, 8].

Functional reduction, obtained through occlusal adjustment and rigid internal fixation, is usually performed through an intraoral approach since it allows adequate fixation, which causes lower morbidity and brings better aesthetic results [7–10]. In contrast to this information, extraoral access was recommended in this situation, since it was necessary to refract the mandibular angle, a procedure that would not be possible through intraoral access, seeing that the perfect visualization of the lingual board is of fundamental importance [11].

There are numerous damages to the immediate reduction of a fracture since its instability propitiates the formation of fibrotic tissues, especially composed of the dense connective tissue of high proliferative power. Also, nonreduction and instability favor the appearance of bone infections, osteomyelitis, either through malnutrition and intimacy of the tissues or through the communication of these fractured stumps to the oral cavity [12].

In the case in question, the formation of a bone callus was noted, shaped as a mass of disorganized tissue, which made its removal possible, although of hard tissue, with ease. To this end, a chisel and hammer were used. The osteotomy was performed, and the poorly positioned stumps were realigned with respect to functional occlusion, seeing that the patient presented malocclusion. It is important to emphasize that this mass of tissue was totally removed to the extent of observing bleeding and healthy tissue [13].

The proposed treatment demonstrated viability and efficacy, correcting aspects involved in the patient's main complaint, such as inadequate occlusion and limitation of mouth opening amplitude, except for loss of sensibility which, due to time, became irreversible.
